# Use of Modified YOLOv5 Algorithm in the Differential Diagnosis of Colonic Crohn's Disease and Ulcerative Colitis on CTE Images

**DOI:** 10.1155/grp/1506567

**Published:** 2025-07-23

**Authors:** Mingbo Bao, Wenjia Liu, Haifeng Shi, Mingzhu Meng, Jian Cao

**Affiliations:** ^1^Department of Radiology, Chinese Medical Hospital of Wujin, Changzhou, China; ^2^Department of Gastroenterology, The Second People's Hospital of Changzhou, The Third Affiliated Hospital of Nanjing Medical University, Changzhou Medical Center, Nanjing Medical University, Changzhou, China; ^3^Department of Radiology, The Second People's Hospital of Changzhou, The Third Affiliated Hospital of Nanjing Medical University, Changzhou Medical Center, Nanjing Medical University, Changzhou, China

**Keywords:** artificial intelligence, diagnosis, inflammatory bowel disease, machine learning

## Abstract

**Background:** Inflammatory bowel disease (IBD) is an immune-mediated disorder characterized by intestinal inflammation and includes two subtypes: Crohn's disease (CD) and ulcerative colitis (UC). The computed tomography manifestations of colonic CD (cCD) and UC are similar, and differential diagnosis is challenging. Our study aimed to investigate the feasibility of using a modified YOLOv5 algorithm for differentiating between cCD and UC on computed tomography enterography (CTE) images.

**Methods:** This multicenter retrospective study analyzed data from a total of 29 cCD patients and 29 UC patients. Five submodels (YOLOv5n, YOLOv5s, YOLOv5m, YOLOv5l, and YOLOv5x) of YOLOv5 were trained and evaluated on the datasets. The CTE images of the cCD group and UC group were divided into a training set, validation set, and test set at a ratio of 8:1:1. Finally, the precision (Pr), recall rate (Rc), and mean average precision (mAP__0.5_ and mAP__0.5:0.95_) of the models were compared.

**Results:** The YOLOv5x model showed the best performance among the five submodels, with mAP__0.5_ of 0.97 and mAP__0.5:0.95_ of 0.97 and 0.84 in the validation set and mAP__0.5_ and mAP__0.5:0.95_ of 0.97 and 0.83 in the test set, respectively. These results demonstrated similar diagnostic accuracy to the two radiologists (84.5%).

**Conclusion:** The modified YOLOv5 algorithm is a feasible approach to distinguish between cCD and UC on CTE images. These findings may facilitate the early detection and differential diagnosis of IBD.

## 1. Introduction

Over the last few years, the prevalence and incidence of inflammatory bowel disease (IBD), including Crohn's disease (CD) and ulcerative colitis (UC), have steadily increased, posing a substantial social and economic burden on governments and health systems. [1–4] This growing concern has attracted the attention of clinicians and radiologists. CD can be subdivided into four types based on the location: ileal CD, colonic CD (cCD), ileocolonic CD, and isolated upper CD [5]. Among them, cCD accounts for 22%~35% of all IBD cases according to the reported literature [6, 7]. However, the CT manifestations of cCD and UC are similar, and differentiating between the two conditions remains a challenge.

Artificial intelligence (AI) has been increasingly adopted across different areas of medicine, with a growing body of research exploring its application in gastroenterology [8]. The clinical use of AI has been primarily aimed at exploring the causes, diagnosing, and predicting outcomes for patients with IBD [9–13]

Sutton et al. [14] investigated the use of deep learning (DL) algorithms to differentiate UC from other intestinal diseases and evaluate the severity of UC during endoscopy. The team analyzed a dataset containing 851 images of UC patients and conducted a five-fold cross-validation. The results revealed that the highest accuracy (87.50%) and area under the curve (AUC) (0.90) were achieved using the DenseNet121 architecture, compared to 72.02% and 0.50 by predicting the majority class (“no skill” model). Additional research into the automatic evaluation of UC endoscopic imagery has highlighted the benefits of DL techniques. A study by Takenaka et al. utilized a DL-based model to assess endoscopic images from UC patients [15]. Their approach achieved a high degree of accuracy (90.1%) in analyzing endoscopic images from 40,758 UC patients in endoscopic remission, all of whom had been assigned a UC Endoscopic Severity Index Score (UCEIS) of 0. The availability of extensive datasets has facilitated the development of innovative solutions to address needs in the management of IBD patients. Nonetheless, the diversity of AI methods, data sources, and clinical outcomes presents challenges in seamlessly integrating AI technology into daily clinical practice, especially in analyzing CT imaging data in the diagnosis of IBD.

The 5th edition of You Only Look Once (YOLOv5), which is a cutting-edge, state-of-the-art computer vision model developed by Ultralytics, is capable of performing both object detection and classification tasks simultaneously. In this paper, YOLOv5 was adapted to detect and classify cCD and UC on computed tomography enterography (CTE) images.

## 2. Patients and Methods

### 2.1. Patients

**Figure 1:**
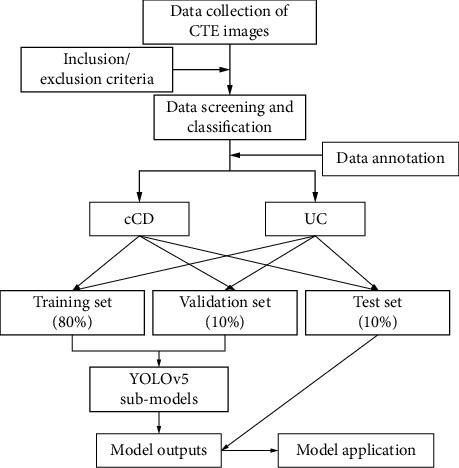
Flow chart of this study.

Ethics approval was obtained from the Second Hospital of Changzhou Affiliated with Nanjing Medical University (Ethics Number: [2022] KY224-01). This retrospective study analyzed data from two independent hospitals in Jiangsu, China, namely, The Affiliated Changzhou No. 2 People's Hospital of Nanjing Medical University and the Chinese Medical Hospital of Wujin. All patients had complete clinical and CTE data. A total of 29 cCD patients (21 males; 8 females) and 29 UC patients (18 males; 11 females) were included. Exclusion criteria are as follows: (1) patients in the remission phase; (2) CTE images with poor quality. A flow chart of the study is shown in Figure 1.

### 2.2. Bowel Preparation Before CTE

Patients were instructed to consume a low-residue diet and remain fasting after the evening meal on the day before the examination. In addition, oral magnesium was administered half an hour after the evening meal. The patients fasted (refrained from eating and drinking) before the CT examination in the morning. A hypotonic solution was diluted with mannitol to prepare an isotonic solution (300 mOsmol). Subsequently, 1000–1500 mL of isotonic solution was ingested approximately 45–50 min before the scan to distend the bowels. Ten minutes before the CT scan, patients were intramuscularly injected with 10 mg of raceanisodamine hydrochloride (1 mL:10 mg), and 500 mL of isotonic solution was ingested again.

Attention: (i) The amount of isotonic solution was modulated to reach the appropriate dosing in pediatric and elderly patients. (ii) Oral isotonic solution was not required for patients with complete intestinal obstruction, as the intestinal fluid was sufficient for intestinal distension. (iii) Raceanisodamine hydrochloride is contraindicated in patients with prostatic hypertrophy, glaucoma, and arrhythmia.

### 2.3. CT Scan Protocols

Two different models of CT scanners were used: a General Electric CT scanner (Discovery CT750 HD) and a Siemens CT scanner (Somatom Definition Flash CT, Germany). The CT scans (tube voltage of 100~120 kV, tube current modulated automatically, pitch of 1.1, slice thickness of 5 mm, layer spacing of 3 mm, 512∗512 matrix, and field of view of 70 cm) included the abdomen and pelvis from the dome of the diaphragm to the pubic symphysis; reformatted sagittal and coronal CT images were generated. Specifically, 100 mL of nonionic iodinated contrast material (320 or 350 mg I/mL) was injected through the elbow vein at a rate of 4.0 mL/s. Enhanced CT scans started at 45 s after the injection of the contrast agent.

### 2.4. Image Analysis

Two radiologists, each with more than 10 years of abdominal CT experience, assessed the CTE images through the picture archiving and communication systems (PACSs) (http://www.jinpacs.com/) independently. The diagnosis of IBD was based on CTE signs of active IBD such as wall thickness of greater than 3 mm in a distended bowel loop, mural hyperenhancement (increased attenuation of the inner layer), mural stratification (bilaminar or trilaminar appearance of the bowel wall), prominence of the vasa recta (comb sign), and mesenteric lymph nodes [16, 17]. The analysis was conducted in two stages. In Stage 1, the two radiologists were blinded to the patients' histopathological diagnoses and performed the diagnostic assessment. In Stage 2, the CTE images were assessed again after unmasking the histopathological diagnosis, and the CTE images were saved at this stage. Disparities between the two radiologists were resolved by discussion or by a third senior radiologist.

### 2.5. CTE Image Dataset and Data Labeling

**Figure 2:**
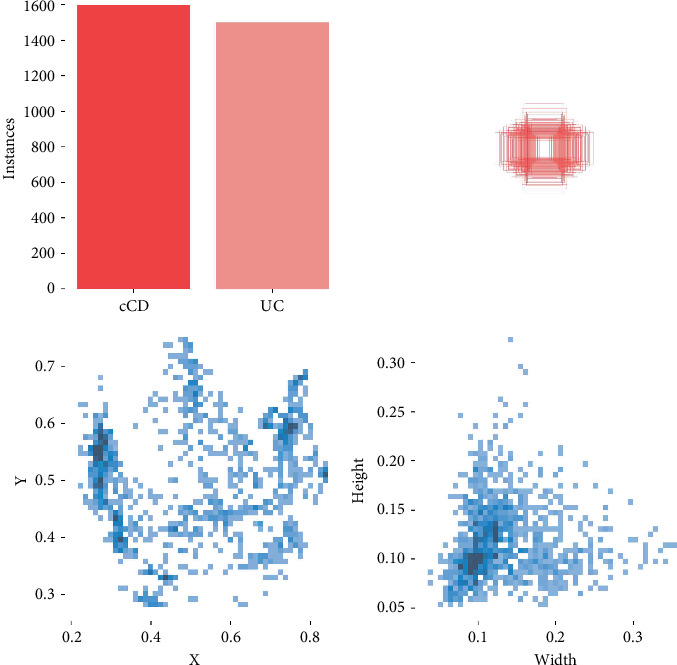
Distribution plot of the labeled data. *X*, *Y*: label coordinates in the CTE images. Width, height: width and height of the label. Instances: the number of labels. cCD and UC refer to the cCD group and UC group, respectively. A darker color indicates more data.

Finally, 1290 cCD images (cCD group) and 982 UC images (UC group) were obtained. Image annotation (YOLO format) was performed using the LabelImg offline software package (Tzutalin, Version 1.8.1, available at https://github.com/tzutalin/labelImg). Annotations completed by one radiologist were verified by the other radiologist. The data of the two groups were randomly (Simple random method) divided into a training set, validation set, and test set at a ratio of 8:1:1. Labeled data distribution is shown in Figure 2.

### 2.6. Computer Configuration

The computer environment consisted of Windows 10 Enterprise 64-bit, Intel(R) Core (TM) i7-10700F 2.9GHz, 32 GB RAM, and NVIDIA GeForce RTX 2060 GPU with 12 GB memory. Data were stored on a hard disk (512 GB solid-state drive) for offline analysis. The models were built based on Python (Version, 3.8), Pytorch (Version, 2.0.1), and CUDA 11.8. While running the model, all other programs were closed.

### 2.7. The Architecture of YOLOv5

**Figure 3:**
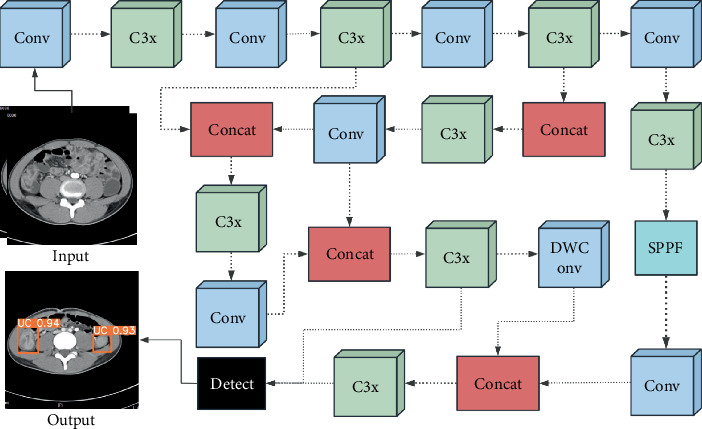
The architecture of YOLOv5, mainly involving the input, calculation process, and output.

The performance of five modified submodels (YOLOv5n, YOLOv5s, YOLOv5m, YOLOv5l, and YOLOv5x) of YOLOv5 was compared in the same dataset. The architecture of YOLOv5 and modified hyperparameter settings are presented in Figure 3 and Table 1. ReLU6 and HS were chosen as the activation functions in the convolutional layers; the formulas for the calculation are as follows:

(1) $$\begin{array}{c} \text{ReLU}\left(6\right)=\min\left(\max\left(x,0\right)6\right), \end{array}$$

(2) $$\begin{array}{c} \text{HS}=h-\text{sigmoid}=\frac{\text{ReLU}6\left(x+3\right)}{6}. \end{array}$$

### 2.8. Intersection Over Union (IoU) and Loss Function

**Figure 4:**
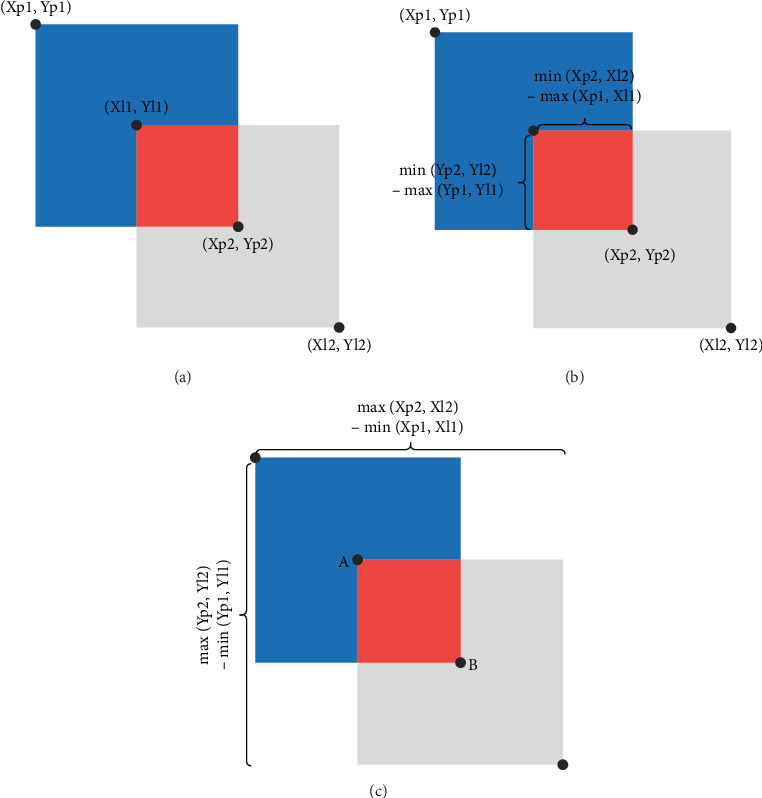
Schematic view of the loss function and IoU/GIoU calculation. (a) The coordinates used to calculate S1 and S2. (b) The calculation method for S1 and IoU. (c) The calculation method for GIoU.

The IoU is calculated by dividing the surface area of the intersection (S1, red area) between two rectangular boxes by the surface area (S2) of the union between those two rectangles. (Xp1, Yp1) and (Xp2, Yp2) represent the top-left and bottom-right corners, respectively, of the predicted rectangular box. (Xl1, Yl1) is the top left corner, and (Xl2, Yl2) is the bottom right corner of the labeled rectangular box (see Figure 4a,b). Formulas for calculating S1, S2, IoU, and loss_IoU_ are as follows:

(3) $$\begin{array}{c} S_{1}=\left(\min\left(\text{Xp}2,\text{Xl}2\right)-\max\left(\text{Xp}1,\text{Xl}1\right)\right)*\left(\min\left(\text{Yp}2,\text{Yl}2\right)-\max\left(\text{Yp}1,\text{Yl}1\right)\right), \end{array}$$

(4) $$\begin{array}{c} S_{2}=\left(\text{Xp}2-\text{Xp}1\right)*\left(\text{Yp}2-\text{Yp}1\right)+\left(\text{Xl}2-\text{Xl}1\right)*\left(\text{Yl}2-\text{Yl}1\right)-S1, \end{array}$$

(5) $$\begin{array}{c} \text{IoU}=\frac{S1}{S2,} \end{array}$$

(6) $$\begin{array}{c} {\text{loss}}_{\text{IoU}}=1-\text{IoU}. \end{array}$$

IoU can have a value between 0 and 1. An IoU value of 0 indicates no overlap between the two rectangular boxes, while an IoU of 1 indicates complete overlap. This study utilized the GIoU algorithm. While IoU only considers overlapping areas, GIoU includes nonoverlapping areas as well, providing a more accurate assessment of the degree of coincidence (Figure 4c). The formula for calculating GIoU was defined as follows:

(7) $$\begin{array}{c} {\text{S}}_{3}=\left(\max\left(\text{Xp}2,\text{Xl}2\right)-\min\left(\text{Xp}1,\text{Xl}1\right)\right)*\left(\max\left(\text{Yp}2,\text{Yl}2\right)-\min\left(\text{Yp}1,\text{Yl}1\right)\right), \end{array}$$

(8) $$\begin{array}{c} \text{GIoU}=\text{IoU}-\frac{\text{S}3-\text{S}2}{\text{S}3}. \end{array}$$

An IoU of 0 suggests no intersection between *A* and *B*. However, GIoU can also display negative values, with a value of −1 indicating infinite separation between *A* and *B*. Conversely, a GIoU of 1 indicates complete overlap between *A* and *B*. The GIoU algorithm solves the problem of the IoU value being 0 when there is no intersection between *A* and *B*. Loss_GIoU_ can be calculated as:

(9) $$\begin{array}{c} {\text{loss}}_{\text{GIoU}}=1-\text{GIoU}. \end{array}$$

### 2.9. Metrics for Evaluation

The metrics calculated in this study included [18]: precision (Pr), recall rate (Rc), and mean average precision (AP) (mAP). The mathematical formulations are as follows:

(10) $$\begin{array}{c} \text{Pr}=\frac{\text{TP}}{\text{TP}+\text{FP}}, \end{array}$$

(11) $$\begin{array}{c} \text{Rc}=\frac{\text{TP}}{\text{TP}+\text{FN}}, \end{array}$$

(12) $$\begin{array}{c} \text{mAP}=\frac{1}{N}\overset{n}{\underset{i=1}{\sum }}{AP}_{i}. \end{array}$$

True positives (TPs) and true negatives (TN) represent samples that were correctly identified as positive and negative, respectively. False positives (FP) and false negatives (FN) are instances of samples that were incorrectly identified as positives or negatives, respectively. As a two-axis mapping, AP represents the region enclosed by Pr and recall as a function of class for a given class [19]. An essential parameter for network model training is the mAP, which is derived from the mean average Pr for each category.

### 2.10. Statistical Analysis

Statistical analysis was performed using SPSS 16.0 statistical software (IBM). For variables conforming to a normal distribution (Kolmogorov–Smirnov test), the count data were expressed in the form of mean ± standard deviation, and one-way analysis of variance (ANOVA) was used to analyze the variance between the two groups. The Mann–Whitney *U* test was performed if the data did not conform to a normal distribution. *p* < 0.05 was considered statistically significant.

## 3. Results

### 3.1. Age Distribution

A total of 29 cCD patients (mean age 45.31 ± 20.37) and 29 UC patients (mean age 48.10 ± 18.94) were included. The Kolmogorov–Smirnov analysis demonstrated that age in the two groups conformed to a normal distribution (*p* = 0.147 and 0.109, respectively). No significant difference in age was found between the two groups (*F* = 0.292, *p* = 0.591).

### 3.2. Results of Model Training and Validation

In the interrater reliability analysis, the ICC for annotations by two radiologists was 0.945 (95% CI: 0.943–0.999), indicating excellent agreement. In both the training and validation sets, Pr, Rc, mAP__0.5_, and mAP__0.5:0.95_ gradually increased as the training epoch increased, whereas the loss value gradually decreased.

**Figure 5:**
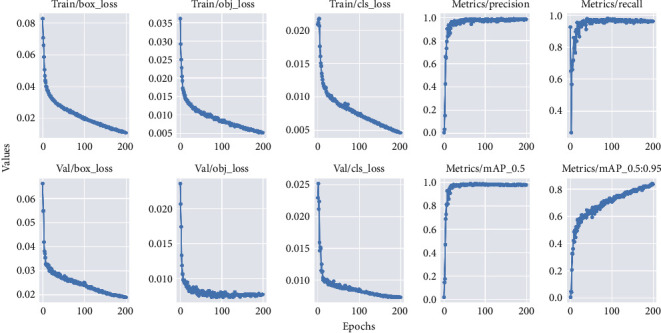
Learning curves of YOLOv5x. Train/Val/box_loss: bounding box detection loss of training set/testing set; Train/Val/obj_loss: object detection loss of training set/testing set; Train/Val/cls_loss: classification loss of training set/testing set. mAP__0.5:0.95_: denotes the mean mAP at the GIoU threshold from 0.5 to 0.95 with a step width of 0.0 (0.5, 0.55, 0.6, 0.65, 0.7, 0.75, 0.8, 0.85, 0.9, and 0.95). mAP__0.5_: mAP with GIoU threshold set at 0.5.

After 200 training epochs, the YOLOv5x model achieved the highest mAP__0.5_ and mAP__0.5:0.95_ of 0.98 and 0.86 in the validation set compared with the other four models (Figure 5). Among all models in our dataset, YOLOv5x exhibited the highest giga floating-point operations per second (GFLOPs) and the time used for training, at 142.1 and 8.14 h, respectively. Detailed data are provided in Table 2.

### 3.3. Visualization

**Figure 6:**
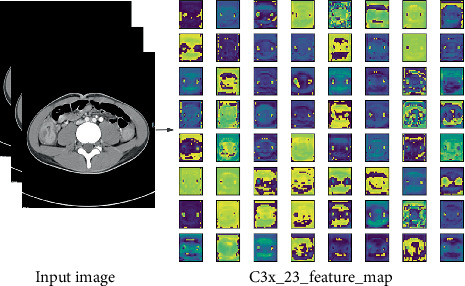
Feature map of a cCD CTE image in the C3x module. Different feature maps from different convolutional layers can be observed in the figure.

Different features were extracted from different convolutional layers in different modules and were observed in the form of pictures, as shown in Figure 6.

### 3.4. Comparison

**Figure 7:**
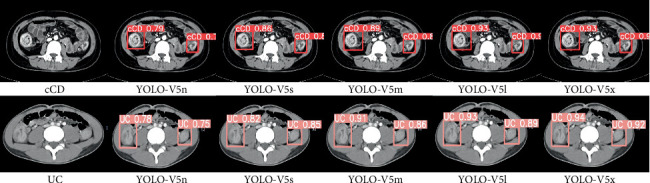
The results of five submodels of YOLOv5 in the detection and classification of cCD (top row) and UC (bottom row) on computed tomography enterography (CTE) images. The CT signs of those two cases were very similar on CTE images. YOLOv5n obtained the lowest confidence score among the submodels, while the confidence score of YOLOv5x was the highest.

During Stage 1, the diagnostic accuracies of radiologists for cCD and UC were 0.79 (23/29) and 0.90 (26/29), respectively. About 84.5% (49/58) of IBD patients were correctly diagnosed by the two radiologists based on CTE images, and nearly 15.5% (9/58) were misdiagnosed. The CT images of those misdiagnosed cases showed signs that could be interpreted as both cCD and UC, with some cases resembling colon cancer. Hence, differentiating between cCD and UC based on CTE images was challenging, even after involving the third senior radiologist (Figure 7).

In the test set, the YOLOv5n model achieved the lowest Pr (0.93), Rc (0.92), mAP__0.5_(0.94), and mAP__0.5:0.95_(0.68), while the YOLOv5x model obtained the highest Pr (0.97), Rc(0.95), mAP__0.5_(0.97), and mAP__0.5:0.95_(0.83). Specifically, the mAP__0.5:0.95_ of YOLOv5x for cCD and UC were 0.83 and 0.86, respectively. These results are much closer to the diagnostic accuracies of two radiologists. Detailed data are provided in Table 3.

## 4. Discussion

In this paper, five submodels of YOLOv5 were adapted to detect and classify cCD and UC on CTE images. This methodological framework demonstrates potential for characterizing CTE imaging biomarkers in IBD. It has contributed to a better understanding of the pathogenesis of IBD. Our findings revealed that variations in model complexity within the YOLOv5 architecture resulted in different mAP in the differential diagnosis of cCD and UC on CTE images, with mAP increasing with model complexity. The YOLOv5x model achieved the highest mAP__0.5_ and mAP__0.5:0.95_ of 0.98 and 0.86 in the validation set, respectively. Moreover, similar results were obtained in the test set, achieving an mAP__0.5_ and mAP__0.5:0.95_ of 0.97 and 0.83, respectively. Hence, the trained YOLOv5x model demonstrated a strong generalization ability in our dataset.

At present, the diagnosis and monitoring of IBD have not been standardized. Therefore, the diagnosis of IBD warrants caution and may require amending the diagnoses during follow-up and adapting treatments accordingly. CD can affect any section of the digestive tract and is subdivided into four types according to the Montreal classification [5, 20]. Due to the similar CT manifestations of cCD and UC, differentiating between these two diseases based solely on radiological images is challenging.

Diverse data types can be used for AI analysis of IBD, including endoscopic data, medical imaging examination, histological data, gene sequence, and protein sequence [21–24]. Among these data types, endoscopic and medical imaging examination data are relatively easy to access and are the most frequently used tools for the diagnosis and management of IBD in daily clinical practice. A previous report showed that supervised ML models yielded a classification accuracy of 71.0%, 76.9%, and 82.7% based on endoscopic data only, histological data only, and combined endoscopic/histological data, respectively. The combined model also obtained an accuracy of 83.3% on the test set [25]. Currently, no other studies are available to compare our DL-based research on CTE data. Dhaliwal et al. [26] developed a random forest model based on multimodal data (including digestive endoscopy, pathology, and magnetic resonance images), achieving an accuracy of 97% in distinguishing between cCD and UC, identifying the seven most significant features (three histological features and four endoscopic features). In contrast to the study by Dhaliwal et al., our proposed YOLOv5 model can visually identify cCD and UC directly from CTE images, potentially providing assistance to clinicians in formulating treatment strategies.

DL is a relatively new field compared to traditional ML, especially the application of DL in medical image analysis. DL models require a large amount of heterogeneous training data and employ a convolutional neural network to extract features of input data from all sites automatically, enabling flexible decisions based on the situation [27, 28]. YOLOv5 is a new one-stage object-detection DL algorithm that features a very high speed of detection and accuracy comparable to that of other state-of-the-art architectures. YOLOv5 has several submodels with different parameter configurations.

Our results also indicate that increased model complexity is an effective way to improve the generalization ability of YOLOv5. Meanwhile, model size also increased with increasing model complexity. The comparative analysis revealed that the YOLOv5x model occupied 120.6 MB of memory, representing a nearly 39.8 times larger size than YOLOv5n (3.03 MB). This large size limits its applicability in deploying the models to mobile devices.

In contrast to the diagnostic accuracies of radiologists in the first stage, the mAP__0.5:0.95_ of the YOLOv5x (0.84, validation set) model was close to the diagnostic accuracies of radiologists (84.5%) to some extent. However, due to the small number of cases, this outcome should be confirmed through further studies.

Nevertheless, the limitations of the present study should be acknowledged. First, the relatively small sample size may lead to model overfitting. A total of 58 patients were enrolled, and selection bias was inevitable. This directly contributed to the relatively low mAP of the model. Additional studies with larger cohorts are required to enhance the generalization ability of YOLOv5, and multicenter collaboration is necessary to obtain larger sample sizes, thereby strengthening the applicability of the results. Second, no external validation dataset was established, and the model's robustness requires further verification. Third, since the aim was to distinguish between cCD and UC on CTE images, the intestinal tube was labeled rather than the intestinal wall. In future work, we plan to analyze the intestinal walls of cCD and UC using YOLOv5. Additionally, the interpretability of the YOLOv5 model and the deployment of the model for distinguishing cCD and UC require further investigation.

## 5. Conclusion

Accurate differentiation between cCD and UC is essential for guiding targeted therapeutic strategies in IBD. Our findings suggest that YOLOv5-based models may assist in the differential diagnosis between cCD and UC. This pilot study explores the feasibility of a YOLOv5-based approach to analyze CTE features, with initial findings indicating that such models could help augment the differential diagnosis of IBD subtypes. However, further in-depth research is required to improve the model's robustness and advance its clinical deployment.

## Figures and Tables

**Table 1:** Hyperparameter settings of YOLOv5.

**Parameter**	**Values**
Input size	640 × 640
Epoch	200
Batch size	4
Learning rate	0.0001
Rotation	45°
Image flip up–down	Yes
Image flip left–right	Yes
Mosaic	Yes
Copy paste	Yes

**Table 2:** Comparison of five submodels of YOLOv5 in the validation set.

**Models**	**Pr**	**Rc**	**mAP**_**_0.5**_	**mAP**_**_0.5:0.95**_	**Hours**	**Size**	**GFLOPs**
YOLOv5n	0.94	0.91	0.95	0.65	1.55	3.03 MB	3.2
YOLOv5s	0.94	0.92	0.95	0.72	1.95	11.50 MB	12.5
YOLOv5m	0.95	0.93	0.96	0.78	3.29	31.50 MB	35.7
YOLOv5l	0.96	0.94	0.96	0.82	4.70	66.50 MB	77.2
YOLOv5x	0.97	0.94	0.97	0.84	8.14	120.60 MB	142.1

Hours indicate time used for training the model; size represents the size of the saved model after the training; GFLOPs present the GFLOP, which can be used as a measure of the complexity of an algorithm; mAP__0.5_, mAP__0.5:0.95_: for details, see Figure 5.

**Table 3:** Comparison of five submodels of YOLOv5 in the test set.

**Models**	**Pr**	**Rc**	**mAP**_**_0.5**_	**mAP**_**_0.5:0.95**_
YOLOv5n	0.93	0.92	0.94	0.68
YOLOv5s	0.94	0.92	0.95	0.73
YOLOv5m	0.94	0.93	0.95	0.79
YOLOv5l	0.96	0.94	0.96	0.82
YOLOv5x	0.97	0.95	0.97	0.83

*Note:* mAP__0.5_, mAP__0.5:0.95_: For details, see Figure 5.

## Data Availability

The data that support the findings of this study are available from the corresponding author upon reasonable request.
